# PEX5R/Trip8b-HCN2 channel regulating neuroinflammation involved in perioperative neurocognitive disorders

**DOI:** 10.1186/s13578-022-00892-6

**Published:** 2022-09-14

**Authors:** Feng Xu, Yafeng Wang, Linlin Han, Daling Deng, Yuanyuan Ding, LuLin Ma, Qingtong Zhang, Xiangdong Chen

**Affiliations:** 1 Department of Anesthesiology, Union Hospital, Tongji Medical College, Huazhong University of Science and Technology, Wuhan, 430022 Hubei China; 2 Department of Anesthesiology, Affiliated Jinling Hospital, Medical School of Nanjing University, Nanjing, 210018 China

**Keywords:** Microglia, Neuroinflammation, Sevoflurane, HCN2, Perioperative neurocognitive disorders

## Abstract

**Background:**

Clinical and animal studies demonstrated that neuroinflammation from anesthesia (sevoflurane) is the main contributor to cause perioperative neurocognitive disorders (PND). Recently, it was reported that microglia respond to hyperpolarization-activated cyclic nucleotide-gated (HCN) channels, which was the target of sevoflurane. Whether HCN channels are involved in the induction of neuroinflammation after sevoflurane exposure is still unclear.

**Results:**

Sevoflurane exposure had increased cognitive dysfunction and anxiety-like behaviors in rats. Rats inhaled with sevoflurane had activated microglia and increased neuroinflammation (IL-1β, IL-6, and TNF-α) in the hippocampus. RNA sequencing identified 132 DEGs (86 up-regulated and 46 down-regulated DEGs [differentially expressed genes]) in the hippocampus of PND rats. RNA-sequencing also uncovered that sevoflurane exposure down-regulates HCN2 expression. Pathway and process enrichment analysis suggests DEGs are mainly enriched in regulation of system process, positive regulation of glutamate secretion, secretion, regulation of synaptic transmission, regulation of nervous system process, behavior, negative regulation of sodium ion transport, and learning or memory. We validated that sevoflurane exposure can down-regulate the levels of PEX5R/Trip8b (an interaction partner and auxiliary subunit of HCN channels) and HCN1-4 channels in the hippocampus of PND rats. We used immunofluorescence staining to identify that HCN2 co-labels with neurons (Neun), astrocytes (GFAP), and microglia (iba1). We observed that the co-labeling of HCN2 with neurons or microglia decreased in the hippocampus and cortex after sevoflurane exposure. Blocking HCN2 by ZD7288 treatment further activated microglia and aggravated sevoflurane exposure-induced anxiety-like behavior, cognitive impairment, and neuroinflammation.

**Conclusions:**

We concluded that sevoflurane exposure can induce an increased level of neuroinflammation, microglial activation, cognitive dysfunction, and anxiety-like behaviors in rats. HCN2 channel, as the target of sevoflurane action, mediates this process. HCN2 might be a target for the treatment and prevention of sevoflurane-induced PND.

**Supplementary Information:**

The online version contains supplementary material available at 10.1186/s13578-022-00892-6.

## Introduction

Perioperative neurocognitive disorders (PND) are common clinical complications in elderly surgical patients after anesthesia [1, 2]. PND is defined as all cognitive impairment that has occurred before surgery, within 30 days after surgery, or between 30 days and 1 year after surgery [2, 3]. It has been reported that the incidence of PND in one week after surgery in elderly patients over 65 years is about 54% [4]. In cardiac surgery patients, its incidence may be as high as 60% [5]. The high incidence of PND makes it an essential topic in perioperative medical research. Its harmfulness and adverse effects on postoperative recovery have seriously increased the burden on the global medical system and the families of surgical patients.

The pathogenesis of PND is complex, involving numerous pathological and molecular biological mechanisms. Many clinical and animal studies have shown that neuroinflammation may play a critical neuropathological process for PND [6–9]. Nevertheless, the mechanisms for the initiation and induction of neuroinflammation after anesthesia are still unclear.

As a homeostasis maintainer in the CNS (central nervous system), microglia is the resident phagocyte in the CNS, which is the first responder to neuroinflammation or injury, and rapidly adjusts its phenotypic and functional changes according to the brain environment [10, 11]. Many published research has reported that microglia is vital in determining neuroinflammatory response in inducing PND [12, 13]. The activation of microglia facilitates the increase of hippocampal neuroinflammatory cytokine levels (interleukin-1β [IL-1β], IL-6, and TNF-α [tumor necrosis factor α]) and finally causes cognitive impairment [14, 15]. In contrast, effectively depleting CNS microglia by using PLX5622 (colony-stimulating factor 1 receptor [CSF1R] inhibitor) reduces hippocampal levels of inflammatory mediators and remarkably improves cognitive impairment [16]. In addition, other animal studies also demonstrated that suppressing microglial activation in CNS may be a therapeutic target for PND caused by anesthesia or surgery [15, 17]. Thus, finding a target that regulates the biological function of microglia is the key to solving PND.

We and others found that sevoflurane, as a volatile anesthetic, can inhibit hyperpolarization-activated, cyclic nucleotide-gated (HCN) channels, and HCN knockout or selective HCN channel blocker ZD7288 can increase anesthetic properties of sevoflurane [18, 19]. Interestingly, an in vitro experiment has provided the novel discovery that HCN channels can be expressed on primary microglia and contribute to various microglial functions and influence the course of microglial activation [20]. However, in vivo research, whether sevoflurane through HCN channels mediates microglial activation and neuroinflammation triggering PND is unknown.

Based on the above evidence, we assumed that sevoflurane exposure can lead to neuroinflammation and the deficit of learning and memory and that these effects are regulated by HCN channels. We used sevoflurane exposure to induce the PND rats model to examine these hypotheses. Furthermore, we employed molecular biology, neurobehavioral science, transcriptome sequencing, etc. Ultimately, in vivo experimental level, our results will provide crucial evidence for the involvement of HCN channels in PND, which might be a therapeutic target for PND treatment.

## Results

### Sevoflurane exposure triggered cognitive impairment and anxiety-like behaviors in rats

**Fig. 1:**
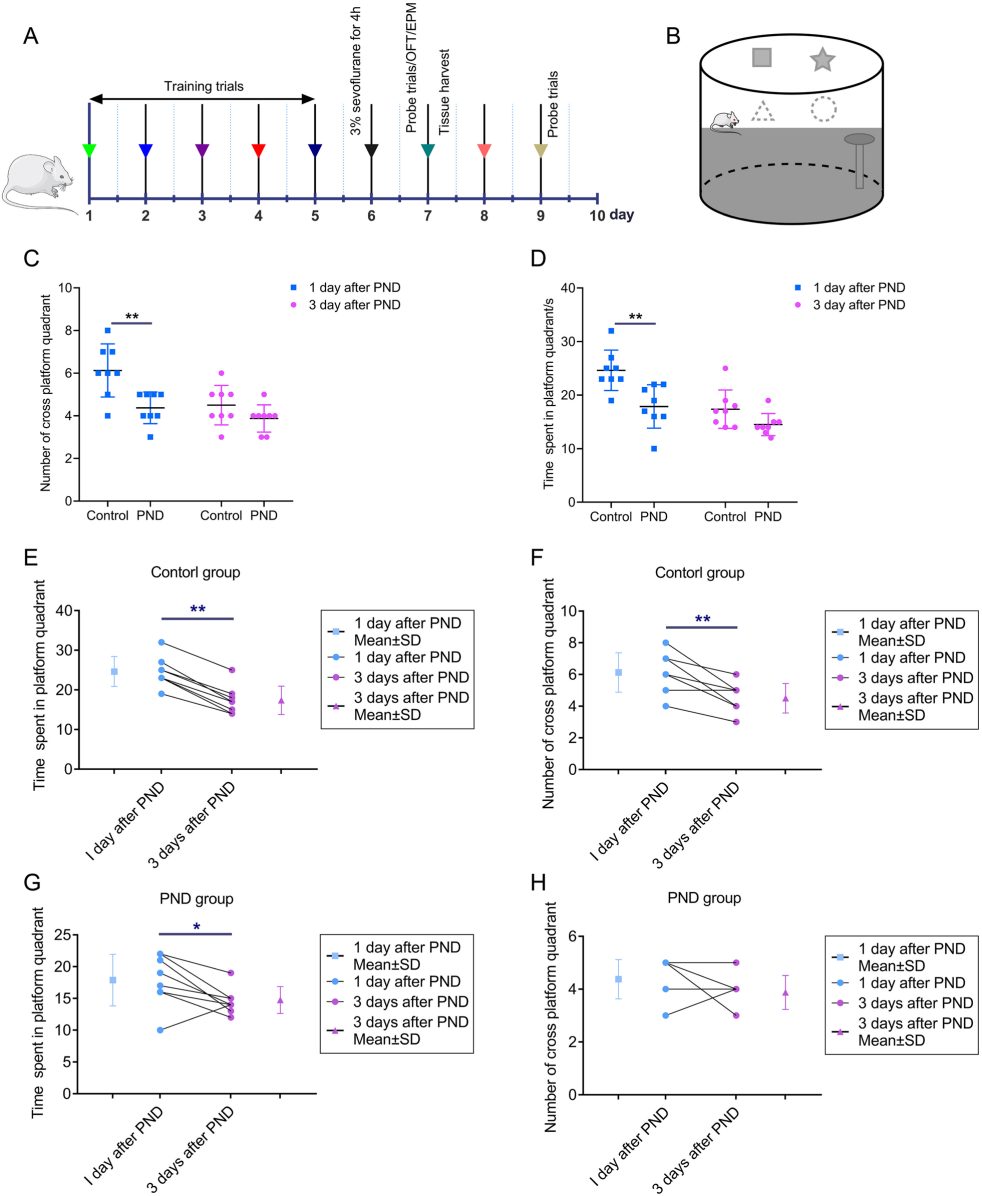
Sevoflurane exposure inducing cognitive dysfunction. **A**, The schedule of the first experiment. **B**, The schematic diagram of the MWM test. **C**, The number of entering the platform quadrant of rats in the MWM test. **D**, The time spent in the platform quadrant of rats in the MWM test. **E**, The time spent in the platform quadrant of rats in the control group on the first day and third day after sevoflurane exposure. **F**, The number of entering the platform quadrant of rats in the control group on the first day and third day after sevoflurane exposure. **G**, The time spent in the platform quadrant of rats in the PND group on the first day and third day after sevoflurane exposure. **H**, The number of entering the platform quadrant of rats in the PND group on the first day and third day after sevoflurane exposure. Data are shown as mean ± SD (n = 8). ^*^P < 0.05 or ^**^P < 0.01.

The schedule of the first experiment and schematic diagram of MWM is shown in Fig. 1A, B. After PND rat models were induced by sevoflurane exposure, PND rats suffered from neurobehavioral changes such as cognitive impairments and anxiety-like behaviors. In probe trials, the number of rats entering the platform quadrant and time spent in the platform quadrant in the PND group was significantly decreased compared with the control group (Fig. 1C, B). On the third day after sevoflurane exposure, the MWM test showed that rats in both PND and control groups had declined as time passed. In the control group, the number of rats entering the platform quadrant and time spent in the platform quadrant evidently decreased on the third day after PND establishment (Fig. 1E, F). Meanwhile, in the PND group, time spent in the platform quadrant of rats was significantly reduced on the third day after PND establishment (Fig 1G). Over time, the rats in both groups showed a natural decline in the memory capacity formed by the water maze training, which contributed to no significant difference in the MWM test between the two groups on the third day after sevoflurane exposure.

**Fig. 2:**
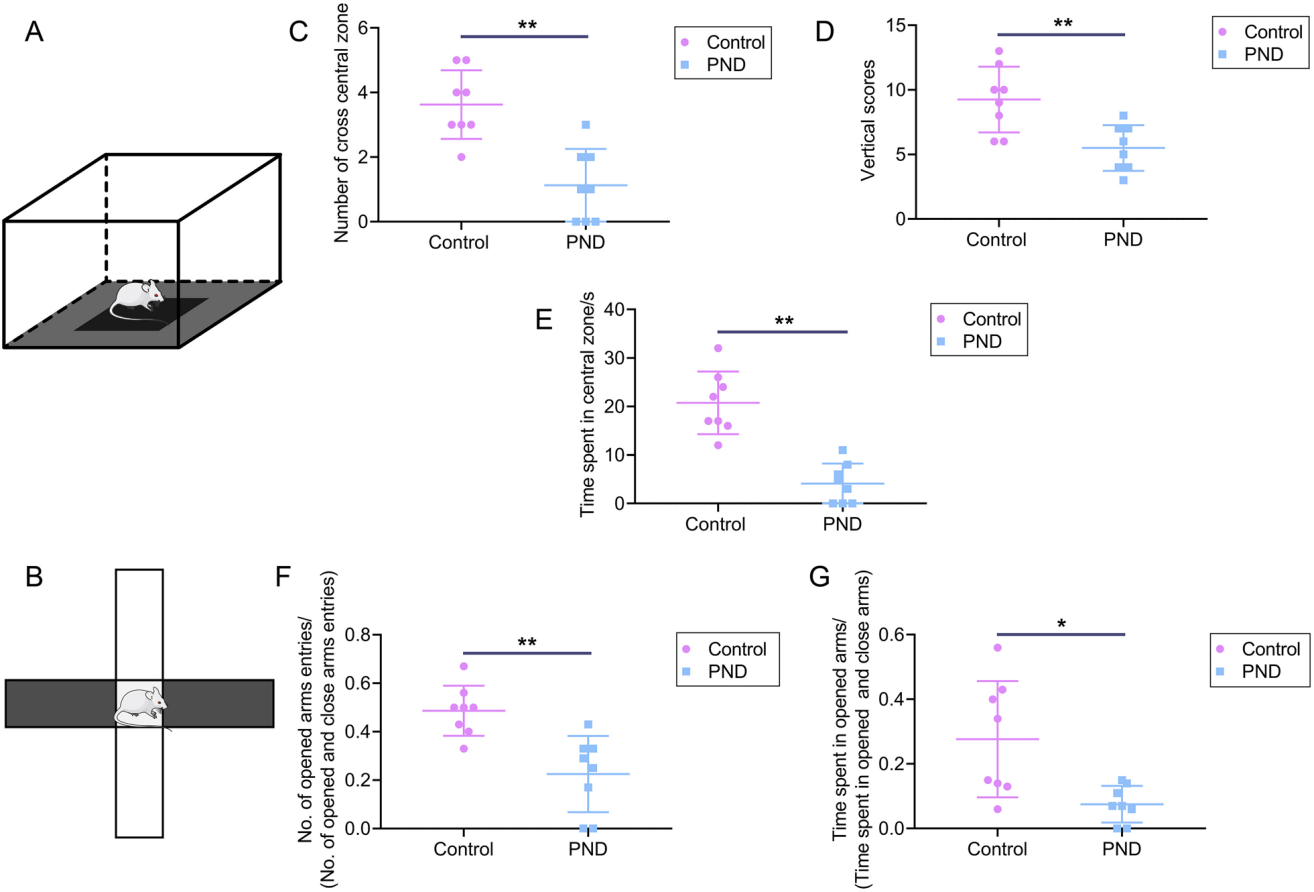
Sevoflurane exposure inducing anxiety-like behaviors. **A**, The schematic diagram of the OFT. **B**, The schematic diagram of the EPM test. **C**, The number of entering the central zone of rats in the OFT test. **D**, The vertical scores of rats in the OFT test. **E**, The time spent in the central zone of rats in the OFT test. **F**, The proportion of opened arms entries of rats in the EPM test. **G**, The proportion of opened arms stays of rats in the EPM test. Data are shown as mean ± SD (n = 8). ^*^P < 0.05 or ^**^P < 0.01.

Furthermore, PND rats also exhibited anxiety-like behaviors, and the schematic diagram of OPT and EPM were also displayed in Fig. 2A, B. The OPT has shown that PND rats had fewer vertical scores, the number of entering the central zone, and time spent in the central zone (Fig. 2C–E). EPM tests have also presented that PND rats had fewer entering the opened arms (Fig. 2F, G). These neurobehavioral experiments showed that sevoflurane exposure induced neurocognitive impairment in rats accompanied by depression and anxiety behaviors.

### The transcriptomic analysis uncovered that sevoflurane exposure down-regulated HCN2 expression. GO enrichment analysis indicated that DEGs were mainly enriched in the regulation of system process, positive regulation of glutamate secretion, secretion, regulation of synaptic transmission, regulation of nervous system process, and learning or memory

**Fig. 3:**
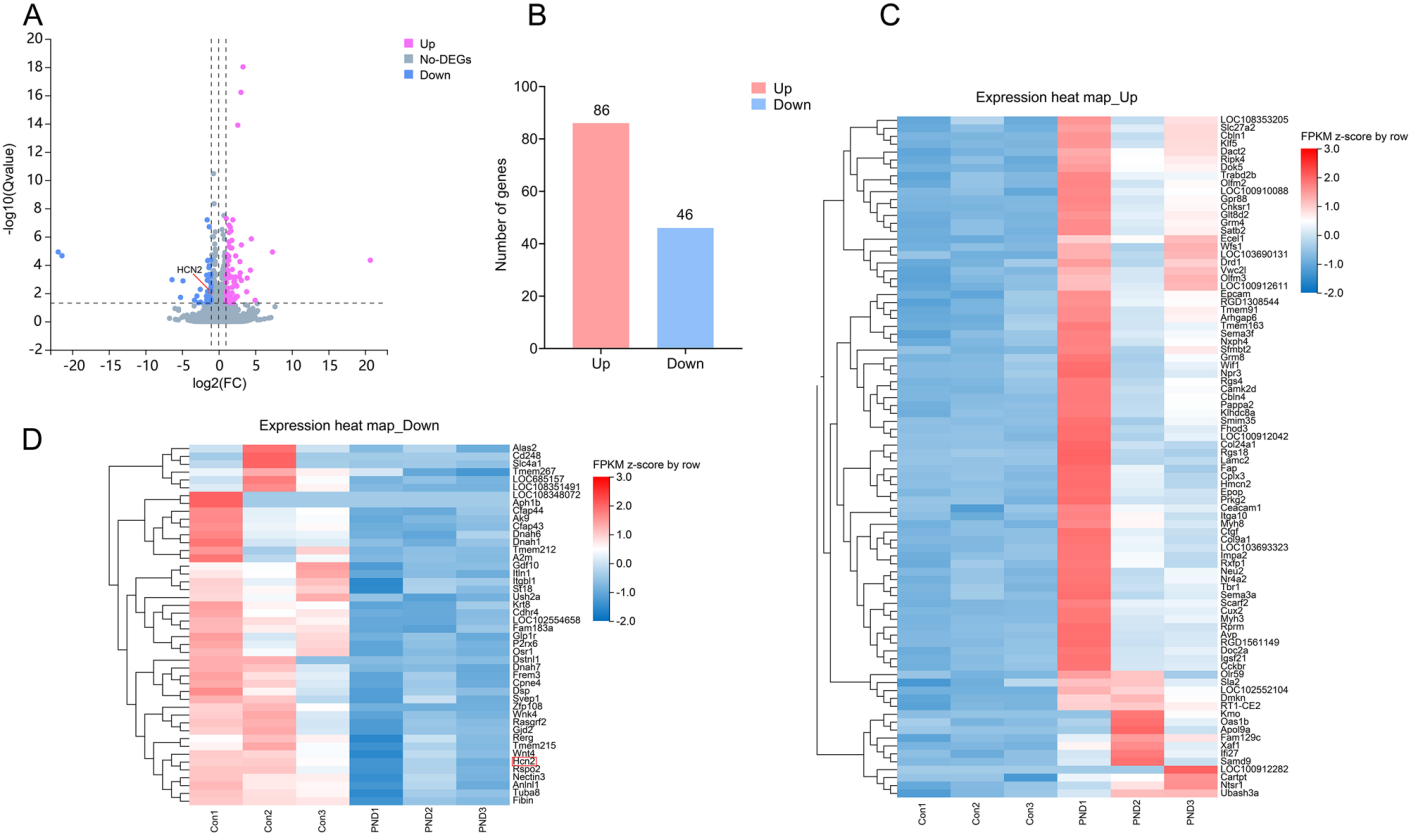
Transcriptomic analysis of the hippocampus in PND rat models. **A**, Volcano plot of differentially expressed genes (DEGs). HCN2, as a down-regulated DEG, was marked in the plot. **B**, the number of down-regulated and up-regulated DEGs. **C**, Up-regulated DEGs cluster heatmaps. **D**, Down-regulated DEGs cluster heatmaps. HCN2, as a down-regulated DEG, was marked in the plot. The horizontal axis is the z-score of the included sample, and the vertical axis is the gene’s name. The warmer the color block is, the higher the expression level is, and the colder the color block is, the lower the expression level is. PND group (n = 3) vs. control group (n = 3), |log_2_(fold change, FC)|> 1 and adjusted *P* < 0.05.

**Fig. 4:**
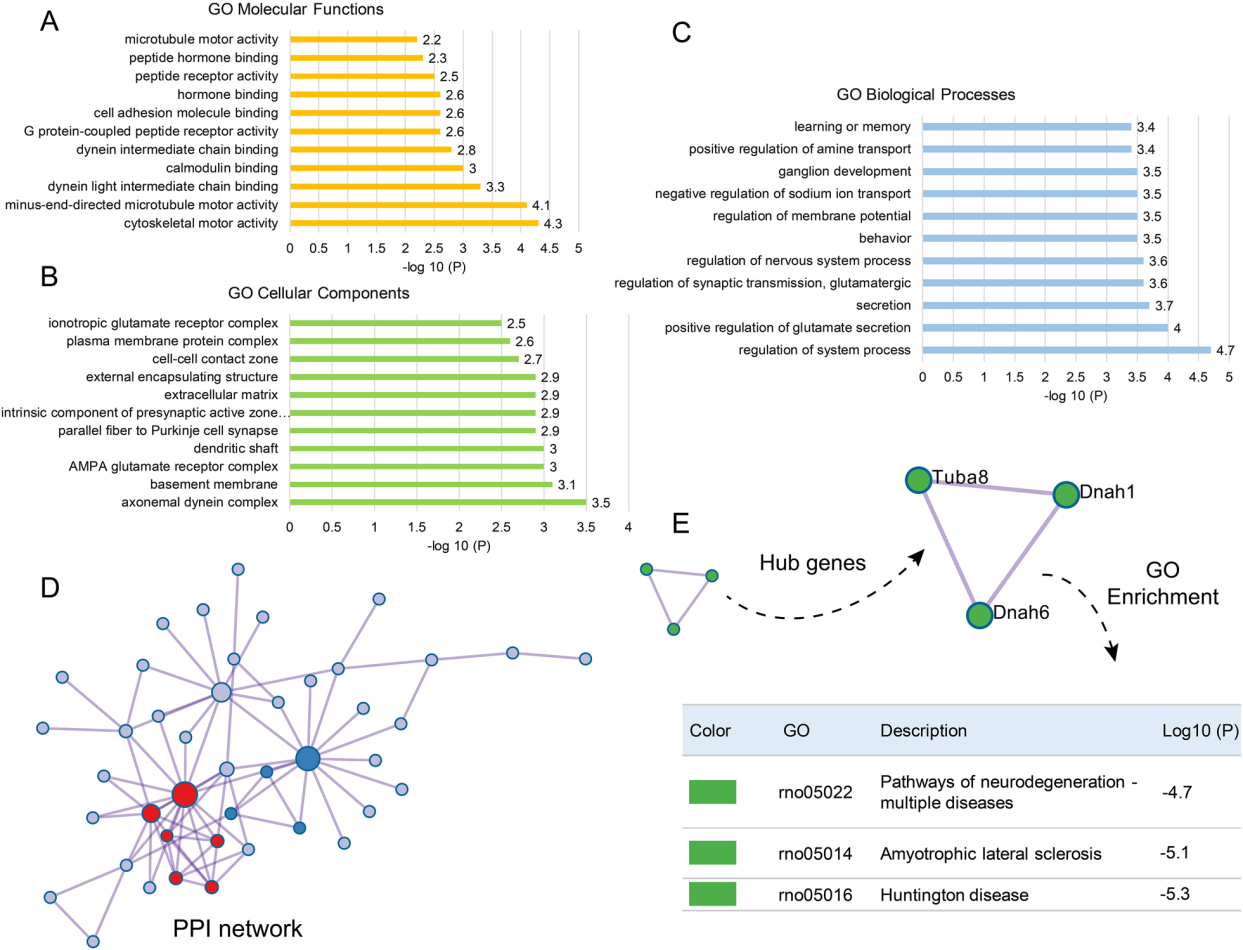
GO enrichment analysis and screening hub genes. **A**, GO molecular Functions enrichment analysis. **B**, GO Cellular Components enrichment analysis. **C**, GO Biological Processes enrichment analysis. From panels A to C, X-axis is log10(P) (the P-value in log base 10) and Y-axis shows enriched terms (one per cluster). Larger log10 (P)-values represent more meaningful enrichment entries. **D**, Protein–protein interaction network and MCODE components in the gene lists. **E**, Functional description of the hub genes and GO enrichment analysis. PND group (n = 3) vs. control group (n = 3).

To identify possible pathways causing PND, we used RNA sequencing to screen for genes and pathways associated with PND. RNA sequencing identified 132 DEGs (86 up-regulated and 46 down-regulated DEGs, with *P* < 0.05 and |log_2_FC [fold change]|> 1) after inducing PND (Fig. 3A–D). In the volcano plot and expression heat map of DEGs, HCN2 marked in Fig. 3A, D was down-regulated after sevoflurane exposure. GO enrichment analysis indicated that the top 11 clusters with enriched representative terms (one per cluster) were shown in Fig. 4A-C. GO Biological Processes enrichment analysis displayed that DEGs were primarily enriched in the regulation of system process, positive regulation of glutamate secretion, secretion, regulation of synaptic transmission, regulation of nervous system process, behavior, regulation of membrane potential, negative regulation of sodium ion transport, and learning or memory (Fig. 4C). GO Cellular Components enrichment analysis showed that DEGs were mainly enriched in axonemal dynein complex, basement membrane, AMPA glutamate receptor complex, dendritic shaft, and parallel fiber to Purkinje cell synapse (Fig. 4B). GO Molecular Functions enrichment analysis presented that DEGs were primarily enriched in cytoskeletal motor activity, minus-end-directed microtubule motor activity, dynein light intermediate chain binding, and calmodulin binding (Fig. 4A).

Protein–protein interaction network and MCODE components were used to identify the gene lists in Fig. 4D. The functional description of the corresponding components (Fig. 4E) shows sevoflurane exposure induced the change of these hub genes related to pathways of neurodegeneration-multiple diseases, Huntington disease, and amyotrophic lateral sclerosis. The finding suggests that sevoflurane exposure can affect synaptic transmission, secretion, nervous system process, ion transport, and learning or memory. Hub genes enrichment analysis also suggests that sevoflurane anesthesia may be a relevant factor for neurodegeneration-multiple diseases, Huntington disease, and amyotrophic lateral sclerosis. Sevoflurane-induced genetic alteration related to neurodegeneration strongly suggests that there might be the same pathogenic pathway between PND and neurodegeneration, which has clinical relevance for PND patients.

### Sevoflurane exposure down-regulated the levels of PEX5R/Trip8b-HCN channels and activated microglia in the hippocampus

**Fig. 5:**
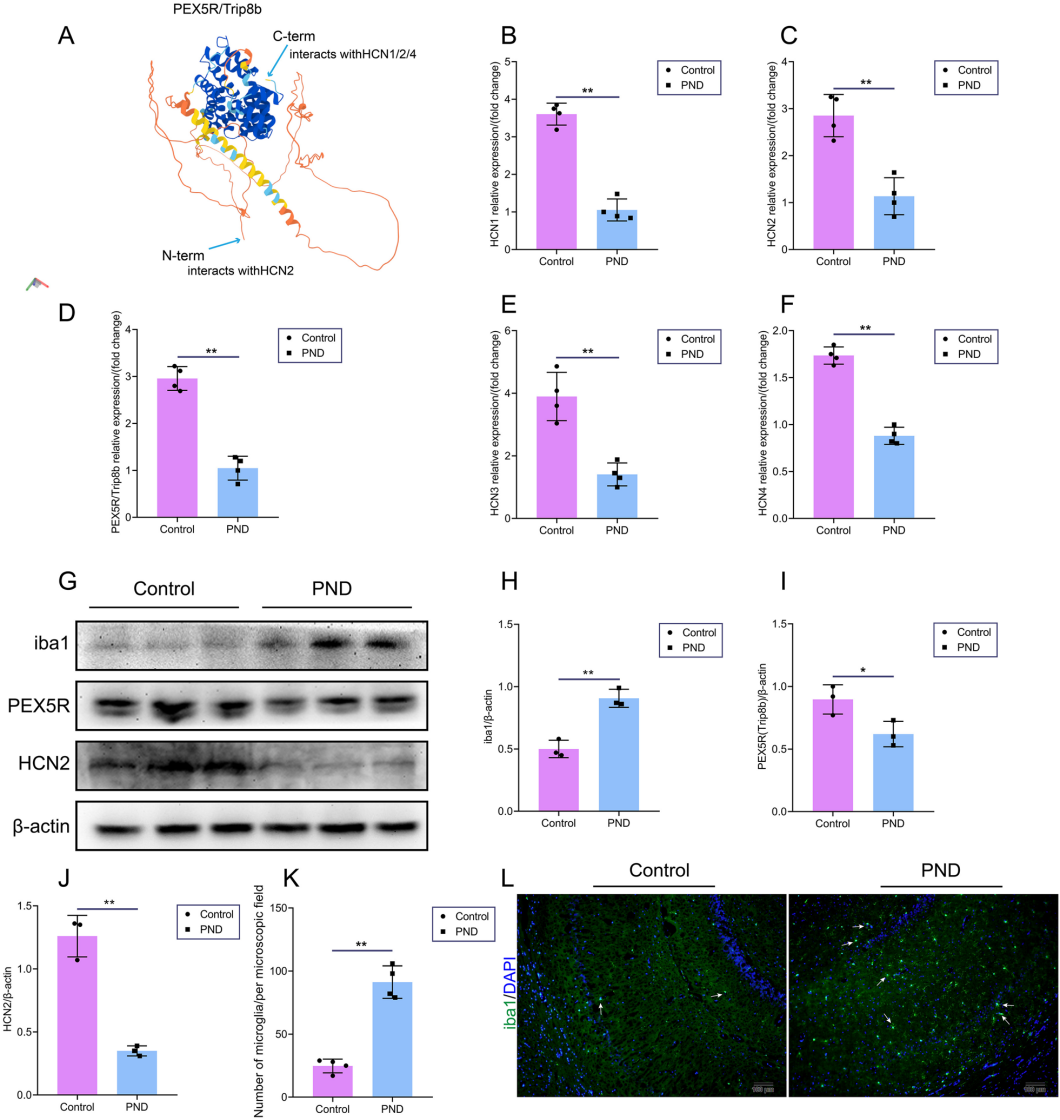
Sevoflurane exposure down-regulating PEX5R/Trip8b and HCN channels and activating microglia. **A**, Structure of PEX5R/Trip8b from UniProt Knowledgebase (https://www.uniprot.org/uniprot/Q925N3), which C and N terminus has a biochemical association with HCN channels. **B**, Sevoflurane down-regulated HCN1 mRNA (n = 4). **C**, Sevoflurane down-regulated HCN2 mRNA (n = 4). **D**, Sevoflurane down-regulated PEX5R mRNA (n = 4). **E**, Sevoflurane down-regulated HCN3 mRNA (n = 4). **F**, Sevoflurane down-regulated HCN4 mRNA (n = 4). **G**, Immunoblotting of iba1, PEX5R, and HCN2 in the hippocampus (n = 3). **H**, Sevoflurane up-regulated iba1 expression. **I**, Sevoflurane down-regulated PEX5R expression. **J**, Sevoflurane down-regulated HCN2 expression. **K**, **L**, Sevoflurane exposure activated microglia (n = 4). Arrows indicate microglia. Data are shown as mean ± SD. ^*^P < 0.05 or ^**^P < 0.01.

According to RNA-sequencing results, regulation of system process, secretion, negative regulation of sodium ion transport, regulation of synaptic transmission, and regulation of nervous system process may be involved in the pathogenesis of PND. We seek to explore molecular evidence linking these pathogenic pathways/processes to PND. Based on our previous research and other reports, HCN is associated with the sodium or potassium ion transmembrane transport, regulating neuronal excitability and transmission [19, 21, 22], and is also expressed in microglia and regulates microglial function [20]. In vitro, HCN2 expressed with a higher degree was found in primary microglia, and regulation of HCN2 can alter the secretion and functions of microglia [20]. In this study, sevoflurane exposure can significantly downregulate the expression of HCN1-4 mRNA (Fig. 5B-F). The tetratricopeptide repeat-containing Rab8b-interacting protein (Trip8b or PEX5R) is an interaction partner and auxiliary subunit of HCN channels [23, 24]. PEX5R/Trip8b C and N terminus have a biochemical association with HCN2 channels (Fig. 5A) [25–27]. Our results also found that sevoflurane exposure reduces the PEX5R/Trip8b and HCN2 expression at the transcript and protein levels (Fig. 5C, D, I, J). Meanwhile, we observed that microglia in the hippocampus also was activated, with the upregulation of iba1 (the marker for microglial activation), as shown in Fig. 5G, H. Many microglia infiltrating the hippocampus were also observed, as shown in Fig. 5K, L. These results indicated sevoflurane exposure reduced levels of PEX5R/Trip8b and HCN channels and activated microglia in the hippocampus.

### HCN2 was expressed in neurons, astrocytes, and microglia. Sevoflurane exposure triggered the down-regulation of HCN2 in microglia and neurons

**Fig. 6:**
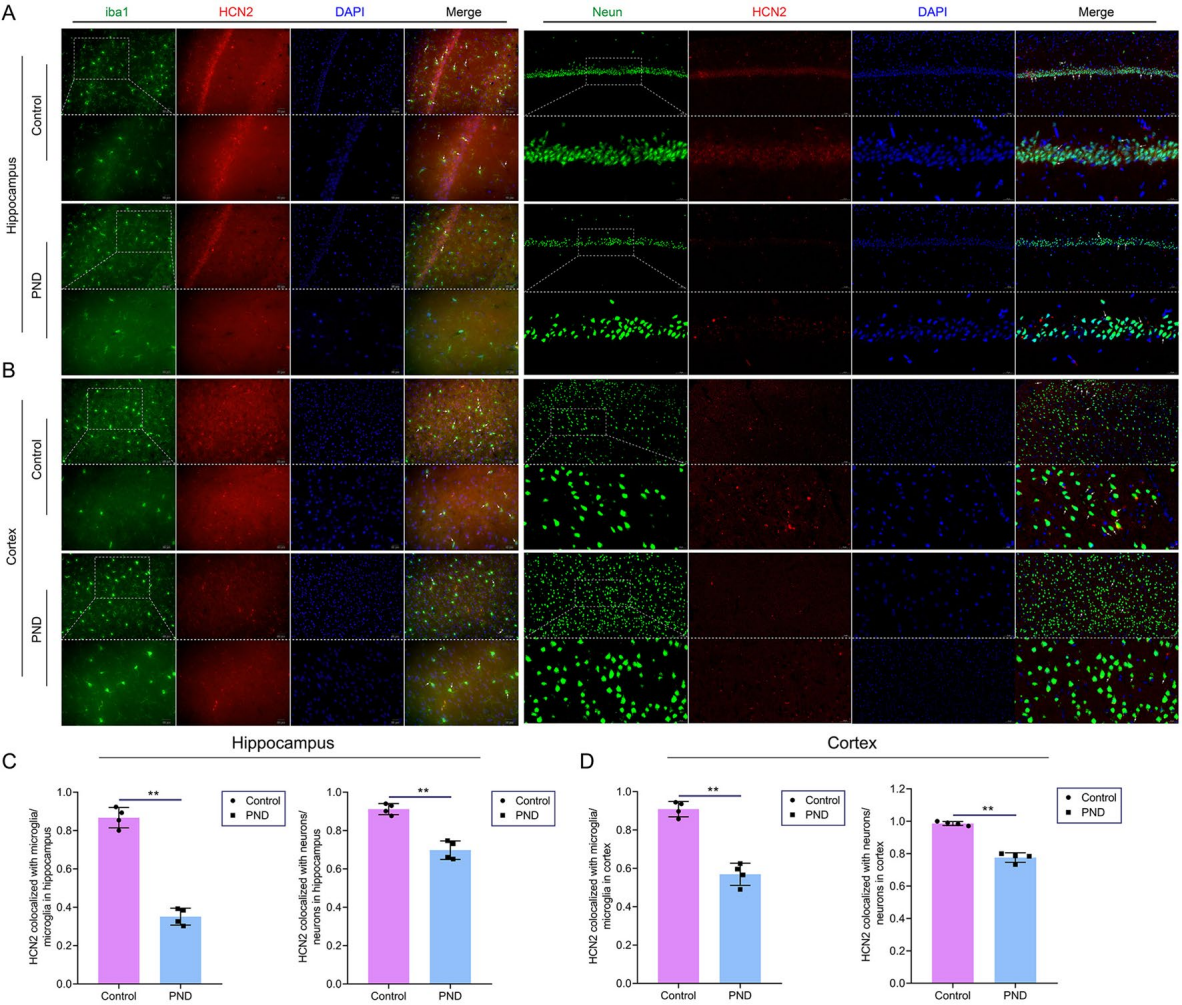
Sevoflurane exposure down-regulating HCN2 in microglia and neurons in the hippocampus and cortex. **A**, Co-labeling of HCN2 with microglia or neurons in the hippocampus. Arrows indicate co-labeling of HCN2 with microglia or neurons. Microglia were labeled with iba1, and neurons were labeled with Neun. **B**, Co-labeling of HCN2 with microglia or neurons in the cortex. Arrows indicate co-labeling of HCN2 with microglia or neurons. **C**, The number of HCN2 co-labeled with microglia or neurons in the hippocampus (n = 4). **D**, The number of HCN2 co-labeled with microglia or neurons in the cortex (n = 4). Data are shown as mean ± SD. ^*^P < 0.05 or ^**^P < 0.01.

We used IF staining to identify that HCN2 co-labels with neurons (Neun), astrocytes (GFAP), and microglia(iba1), which illustrates that HCN2 is widely expressed in different nerve cells of the CNS (Additional file 1: Fig. S1). Then, we found that the co-labeling of HCN2 with microglia reduced in the hippocampus and cortex (Fig. 6A-D). In addition, we also observed that the co-labeling of HCN2 with neurons decreased in the hippocampus and cortex (Fig. 6A-D). Thus, sevoflurane exposure caused extensive down-regulation of HCN2, including on microglia and neurons.

### Sevoflurane exposure caused neuroinflammation in the cortex and hippocampus

**Fig. 7:**
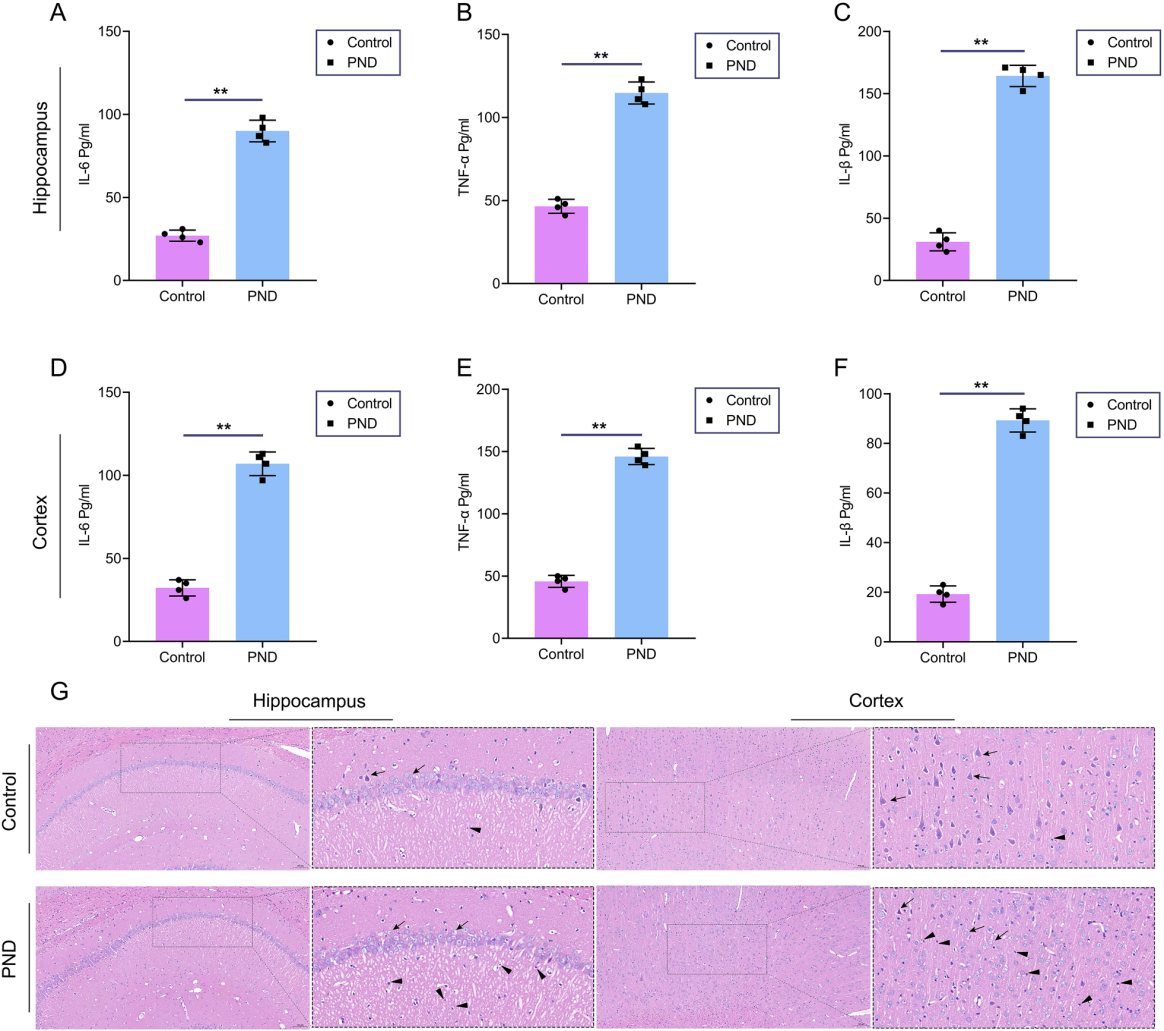
Sevoflurane inducing PND with neuroinflammation in the cortex and hippocampus. **A**, Sevoflurane up-regulated IL-6 in the hippocampus (n = 4). **B**, Sevoflurane up-regulated TNF-α in the hippocampus (n = 4). **C**, Sevoflurane up-regulated IL-1β in the hippocampus (n = 4). **D**, Sevoflurane up-regulated IL-6 in the cortex (n = 4). **E**, Sevoflurane up-regulated TNF-α in the cortex (n = 4). **F**, Sevoflurane up-regulated IL-1β in the cortex (n = 4). **G**, HE staining of cortex and hippocampus. Arrows indicate neurons. Triangles indicate inflammatory cells. Data are shown as mean ± SD. ^*^P < 0.05 or ^**^P < 0.01.

After inducing PND, we used ELISA and HE staining to estimate neuroinflammation. In HE staining, we have found that neurons were intact without morphological alteration, and there are some inflammatory cells (small round nuclei) infiltrating the hippocampus and cortex of PND rats (Fig. 7G). ELISA also displayed that proinflammatory cytokines (IL-6, IL1β, and TNFα) were apparently increased in PND rats' cortex and hippocampus compared with the control group (Fig. 7A, F). This evidence displayed that sevoflurane exposure triggered a neuroinflammatory response.

### ZD7288 treatment aggravated sevoflurane exposure-induced cognitive impairment and anxiety-like behaviors of PND rats

**Fig. 8:**
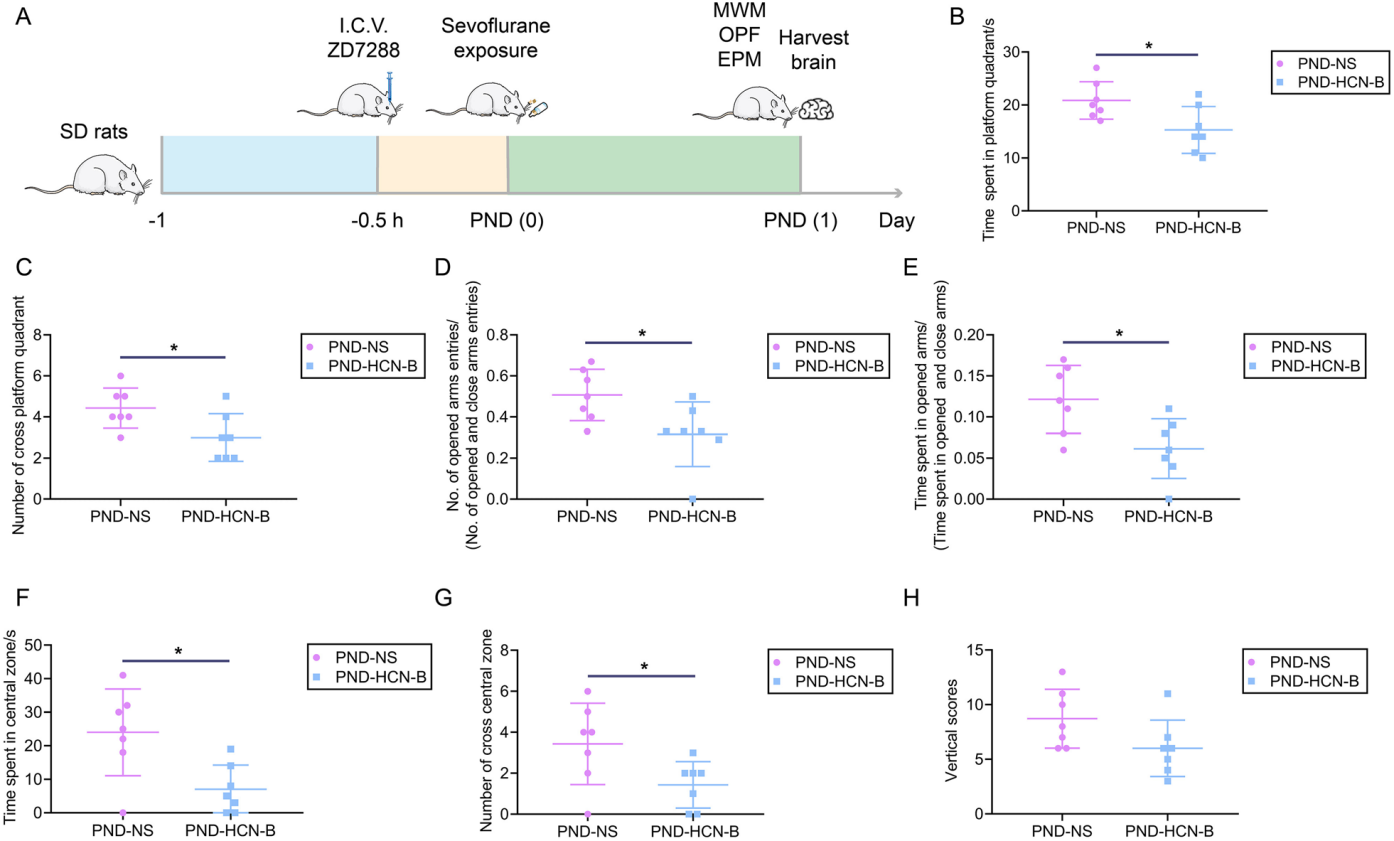
ZD7288 treatment aggravates PND rats’ cognitive impairment and anxiety-like behaviors. **A**, The schedule of the second experiment. **B**, The time spent in the platform quadrant of rats in the MWM test. **C**, The number of entering the platform quadrant of rats in the MWM test. **D**, The proportion of opened arms entries of rats in the EPM test. **E**, The proportion of opened arms stays of rats in the EPM test. **F**, The time spent in the central zone of rats in the OFT test. **G**, The number of entering the central zone of rats in the OFT test. **H**, The vertical scores of rats in the OFT test. Data are shown as mean ± SD (n = 7). ^*^P < 0.05 or ^**^P < 0.01.

The schematic diagram of the second experiment is displayed in Fig. 8A. ZD7288 is a selective and effective HCN channel blocker, which was harnessed to inhibit the HCN channel in the brain. After blocking HCN channels in brian, rats in the PND-HCN-B group showed more increased cognitive impairments and anxiety-like behaviors than rats in PND-NS. In probe trials, the number of entering the platform quadrant and time spent in the platform quadrant of rats in the PND-HCN-B group significantly decreased compared with the PND-NS group (Fig. 8B, C) . In evaluating anxiety-like behaviors, OPT has shown that rats in the PND-HCN-B group had fewer entering the central zone and time spent in the central zone (Fig. 8F, G). The EPM test also presented that rats in the PND-HCN-B group had fewer entering the opened arms (Fig. 8D, E). These neurobehavioral experimental results show that blocking HCN2 can aggravate sevoflurane exposure-induced cognitive impairment and anxiety-like behaviors, which presents a synergistic effect.

### ZD7288 treatment reduced HCN2 expression, increased microglial activation, and aggravated sevoflurane exposure-induced neuroinflammation

**Fig. 9:**
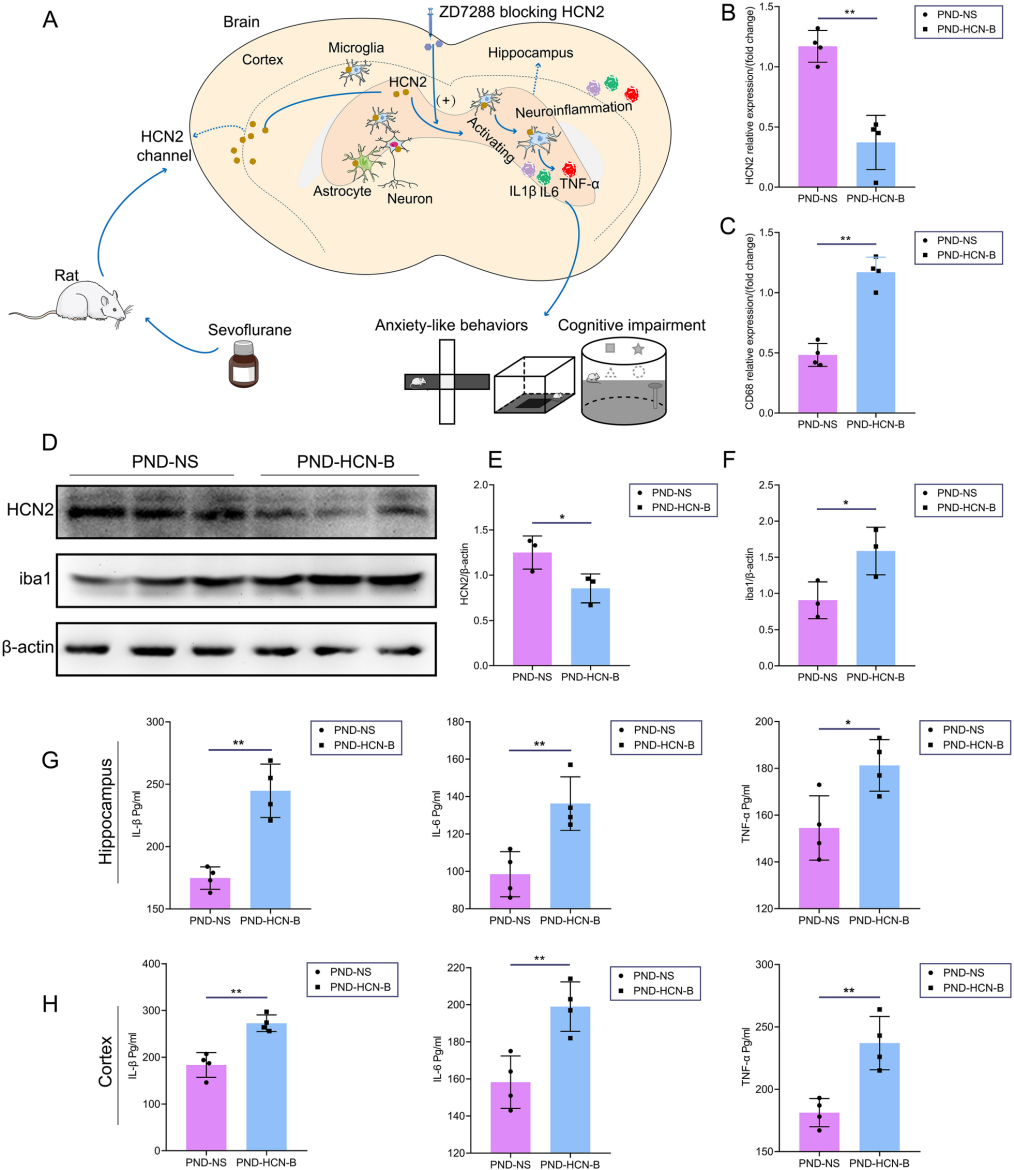
ZD7288 treatment increasing neuroinflammation and microglial activation of PND rats. **A**, Diagrammatic illustration of our findings. HCN2 is extensively expressed in different cells of the cortex and hippocampus, including neurons, microglia, and astrocyte. Sevoflurane down-regulated HCN2 mediating cognitive dysfunction, anxiety-like behaviors, microglial activation, and neuroinflammation. Sevoflurane combined with ZD7288 (HCN2 blocker) produced strong synergies in this process. **B**, ZD7288 treatment down-regulating HCN2 mRNA of PND rats (n = 4). **C**, ZD7288 treatment up-regulating CD68 mRNA of PND rats (n = 4). **D**, Immunoblotting of HCN2 and iba1(n = 3). **E**, ZD7288 treatment down-regulating HCN2 expression of PND rats. **F**, ZD7288 treatment activating microglia of PND rats. **G**, ZD7288 treatment increasing IL-6, IL-1β, and TNF-α in the hippocampus of PND rats (n = 4). **H**, ZD7288 treatment increasing IL-6, IL-1β, and TNF-α in the cortex of PND rats (n = 4). Data are shown as mean ± SD. ^*^P < 0.05 or ^**^P < 0.01.

After ZD7288 preconditioning PND rats, we employed PCR and WB to estimate the HCN2, iba1, and CD68 (indicative of microglial phagocytosis) expression. ZD7288 treatment down-regulated HCN2 expression at the protein level and transcription level (Fig. 9B, E). ZD7288 treatment in PND rats can increase microglial activation in the hippocampus (Fig. 9D, F). Moreover, CD68 mRNA was also up-regulated after blocking HCN2 in the hippocampus (Fig. 9C).

Then, we used ELISA to detect the neuroinflammatory level. In the PND-HCN-B group, ELISA has presented that PND rats’ proinflammatory cytokines (IL-6, IL1β, and TNFα) were significantly increased in both the cortex and hippocampus compared with rats in the PND-NS group (Fig. 9G, F). Therefore, inhibition of HCN2 can further aggravate sevoflurane exposure-induced neuroinflammation and activation of microglia.

## Discussion

Sevoflurane, as a common inhalation anesthetic, is widely used in the field of anesthesiology over the world. HCN channels as the target for sevoflurane were sufficiently established by our team and other researchers [18, 19]. To the best of our knowledge, this study was the first report to demonstrate that sevoflurane acts on its target-HCN2 channel to regulate microglial function and neuroinflammation in vivo, triggering cognitive impairment and anxiety-like behaviors in rats. Our findings have addressed that HCN2 has a significant regulatory effect on microglial activation and neuroinflammation in mediating sevoflurane-induced PND. Moreover, RNA-sequencing uncovered sevoflurane exposure down-regulates HCN2, and pathway and process enrichment analysis suggests DEGs are mainly enriched in regulation of system process, positive regulation of glutamate secretion, secretion, regulation of synaptic transmission, regulation of nervous system process, and learning or memory, which might be associated with PND.

As we know, PND is closely associated with surgery and anesthesia. Our results have illustrated that sole sevoflurane exposure without surgery could induce cognitive dysfunction and anxiety-like behaviors. These behavioral changes are also accompanied by neuroinflammation and microglial activation. Similarly, previous studies also reported that rats only exposed to 2% sevoflurane for five hours or 2.5% sevoflurane for six hours produced cognitive impairment [28, 29]. In this study, rats have exposed to 3% sevoflurane for four hours to establish the PND models, which also caused the cognitive deficiency. Together, we argued that a long time or high dose of sevoflurane exposure could trigger rats’ cognitive dysfunction. It may also suggest that reducing the dosage or the duration of sevoflurane exposure can be beneficial for patients at risk of PND. On the third day after PND, we found that PND rats had no difference in the MWM test compared with rats in the control group, which suggests cognitive impairment induced by sevoflurane was time-dependent, and PND symptoms improved with time delay.

Clinical manifestations of PND include cognitive impairment and confusion, anxiety, and personality changes. Our study used OFT and EPM to estimate anxiety-like behaviors of PND, which thoroughly examined neurobehavioral changes in the PND rats model. In this study, sevoflurane exposure triggered anxiety and decreased space exploration capability, consistent with the previous study [30]. These findings suggest that PND rats with cognitive dysfunction also presented anxiety-like behaviors, which should attract more attention from perioperative medicine.

Neuroinflammation is regarded as a critical role in mediating PND, and reducing the level of inflammatory mediators can ameliorate PND [6, 7, 31]. In our work, sevoflurane exposure triggered CNS inflammation and up-regulated the proinflammatory cytokines of TNF-α, IL-6, and IL-1β. Notably, ELISA found that proinflammatory cytokines in both cortex and hippocampus were all increased after inducing PND rats by inhalation of sevoflurane. As we know, sevoflurane is inhaled through lung tissue into the bloodstream, travels to the CNS, and interacts with different brain regions. Our results and pharmacological mechanism of sevoflurane in CNS strongly implied that sevoflurane-induced CNS inflammation was extensive and not limited to a specific brain region. The previous study also reported that surgery-induced PND models had marked increases in TNF-α, IL-1β, and IL-6 in the cortex and hippocampus [32]. To sum up, both sevoflurane-induced and surgery-induced PND animal models can produce extensive CNS neuroinflammation.

Microglia is a critical regulator in processing CNS neuroinflammatory changes during PND [16, 33, 34]. Effectively depleting CNS microglia in surgery-induced PND models via inhibiting CSF1R reduced hippocampal levels of inflammatory cytokines and mitigated the development of PND [16]. We also confirmed that microglial activation was associated with neuroinflammation, cognitive dysfunction, and anxiety-like behaviors in sevoflurane-induced PND rats. This finding broadly supports that regulating microglia is a determinant for treating sevoflurane-induced PND.

Subsequently, we tried to find the molecules or channels that sevoflurane interacts with that may be involved with the regulation of microglia. In 2020, a crucial study was the first to report the expression of HCN on primary microglia and explore its function in regulating microglia [20], which provided evidence of linking sevoflurane with microglia. Among HCN channels, HCN2 was reported to be expressed to a higher degree than other HCN subunits in primary microglia [20], which is more closely correlated with microglial function. Likewise, we also observed that HCN2 was expressed in microglia, astrocytes, and neurons, which suggests that HCN2, as a channel, is widely present in the CNS. Therefore, our study focused on examining the role of HCN2 in sevoflurane-induced PND. Based on this finding, in this vivo experiment, our results were the first to prove that sevoflurane exposure down-regulated microglial HCN2 channel and activated microglia in the hippocampus. The unexpected finding was that HCN1, HCN2, HCN3, and HCN4 were all down-regulated after sevoflurane exposure.

Furthermore, PEX5R/Trip8b, as an interaction partner and auxiliary subunit of HCN channels [23, 24], was also down-regulated after exposure to sevoflurane. After ZD7288 treatment and down-regulation of HCN2 in the hippocampus of PND rats, we found that microglia were activated. Moreover, CD68, the marker of microglial phagocytosis, was also up-regulated. It means that ZD7288 treatment mediating down-regulation of HCN2 triggers functional change of microglia, which is consistent with in vitro experiments [20]. Furthermore, PND rats with blocking HCN channels in CNS have aggravated inflammatory responses in the cortex and hippocampus. With further aggravating neuroinflammation and increasing the microglia activation, cognitive impairment and anxiety behaviors were even worse. This neurobehavioral change coincided with marked increases in neuroinflammation intensively implies that combined sevoflurane with HCN channels blocker produces strong synergies in causing PND. Accordingly, we deemed that sevoflurane leads to cognitive impairment, anxiety-like behaviors, and neuroinflammation of PND through regulating PEX5R/Trip8b-HCN2 channel interaction.

There are some limitations in this work that need to be addressed. This study only chose animal experiments to verify our hypothesis and has not carried out in vitro experiments. The reason is that it is difficult to simulate the pathogenesis of PND in vitro. In addition, the treatment of cells with inhaled sevoflurane is still controversial. We used MCODE to identify the hub genes related to PND. These genes were enriched in neurodegeneration-multiple diseases. Thus, these hub genes may be involved in the same pathogenic pathway mediating PND and neurodegeneration, which has clinical relevance for PND patients. At last, future work will be performed on exploring some pathogenic pathways in PND from the perspective of neurodegeneration.

## Conclusion

In a word, this study suggests that sevoflurane exposure can induce an increased level of neuroinflammation, microglial activation, cognitive dysfunction, and anxiety-like behaviors in rats. HCN2 channel, as the target of sevoflurane action, mediates this process. HCN2 might be a target for the treatment and prevention of sevoflurane-induced PND. The pathogenesis diagram is shown in Fig. 9A.

## Methods

The animal protocol of this animal study was approved by the Institutional Animal Care and Use Committee at Tongji Medical College, Huazhong University of Science and Technology. All animal experiments were performed in accordance with a guide to animal ethics.

### Animal groups

The 13–16-month-old male SD (Sprague Dawley) rats were purchased from Biont (Wuhan, China). To verify our hypothesis, we carried out two animal experiments. In the first experiment, the SD rats were randomly assigned to the control group and the PND group (PND). In the PND group, rats have exposed to 3% sevoflurane for four hours to induce PND. Rats in the control group were exposed to oxygen for four hours.

In the second experiment, the PND rats were randomly divided into the following two groups: PND-NS (normal saline) and PND-HCN channels blocker (PND-HCN-B) group. In the PND-HCN-B group, PND rats received a stereotactic intracerebroventricular injection of ZD7288 (HCN channels blocker). In the PND-NS group, PND rats received a stereotactic intracerebroventricular injection of normal saline as solvent control.

### PND rat model

In terms of PND models, the establishment of PND models is mainly divided into anesthesia-induced PND and surgery combined anesthesia-induced PND [28, 35, 36]. In order to precisely explore the effect of sevoflurane on HCN channels, we chose to induce the occurrence of PND by long-time and high-concentration exposure to sevoflurane. A long time and 3% sevoflurane in 100% oxygen for rat anesthesia have been reported to effectively trigger cognitive impairment in previous studies [37–39]. In brief, at first, rats were placed into an induction box respectively. Then, the vaporizer was used to input sevoflurane into the induction box, and the concentration of sevoflurane in the induction box was detected through an anesthesia monitor. The sevoflurane exposure concentration was set at 3%, and 2L/min pure oxygen was used as the carrier gas. After 3% sevoflurane exposure for four hours, rats were transferred to a resuscitation box filled with fresh oxygen. The indoor temperature was kept in the range of 23–25 °C.

### Intracerebroventricular injections

ZD7288 is a selective HCN channel blocker [40, 41], which was harnessed to inhibit the HCN channel in the brain. During the second experiment, the rats were anesthetized with continuous 3% sevoflurane anesthesia on an animal anesthesia mask and then fixed onto a stereotaxic frame (RWD, China). Rats in PND-HCN-B and PND-NS groups were all placed in a stereotactic head frame for intracerebroventricular injections via a microinjection pump. The injection site was located as: AP (anteroposterior) = 1.2 mm, ML (mediolateral) = 1.8 mm, and DV (dorsoventral) = 4.0 mm. Regarding the dosage of ZD7288 in intracerebroventricular injections, the previous study reported that ZD7288 regulated synaptic transmission and generated a dose-dependent inhibition of LTP induction [42]. In detail, low doses (ranging from 0.5 to 10 μg) of ZD7288 have no effect on basal synaptic transmission (fEPSP), and the high dose of 25 μg ZD7288 significantly suppresses basal synaptic transmission [42]. Accordingly, for the sake of avoiding the impact of ZD7288 on LTP and synaptic transmission, we select the dosage of 10 μg ZD7288 (Sigma-Aldrich, America) for intracerebroventricular injections. Rats in PND-HCN-B received an intracerebroventricular injection of 10 μg ZD7288 in 5 μl sterile saline (2 μg/μl of ZD7288) at 0.5 h before sevoflurane exposure. Additionally, rats in the PND-NS group only received an intracerebroventricular injection of 5 μl sterile saline. PND modeling was performed 0.5 h after intraventricular injection.

### Morris water maze (MWM)

MWM test was employed to examine the cognition of rats, as described by the previous report [43]. Clinically, PND mainly occurs within 1 week after surgery and anesthesia [4]. We used the MWM to track cognitive changes. During all neurobehavioral experiments, experimenters were blind to group assignment and outcome assignment. In brief, rats were given three trails: visible platform, hidden platform, and probe trial. On day 1, each rat was trained to swim 4 times a day with a visible platform in the pool. From day 2 to day 5, each rat was trained to swim 4 times a day with a hidden platform in the pool. During swimming training, If the rat finds the platform before the 60 s cut-off, it is allowed to stay on the platform for 5 s and then return to its home cage. If the rat does not find the platform, it was placed on the platform to stay there for 20 s before returning it to its home cage. During the probe trial, the platform was removed from the pool, time spent in the platform quadrant and the number of entering the platform quadrant for each rat were recorded in the software. The probe trial was performed on the first day and third day after inducing the PND rat model.

### Open field test (OFT)

Exploratory activity, autonomous animal, and anxiety-like behavior were examined by using an open-field apparatus (100 × 100 × 100 cm) [44, 45]. The center area was set as a square 20 cm away from the wall of the open-field apparatus. Each rat was placed in the center area of the open-field apparatus. The time spent in the central area, number of entering the central area, and vertical score (number of the animal standing on both feet) were recorded for 5 min. Since the cognitive tests showed no difference between groups on day 3 after sevoflurane exposure, we focused only on the behavioral change on day 1 after sevoflurane exposure. Thus, the OFT was implemented on the first day after inducing the PND rat model.

### Elevated plus maze (EPM) test

Anxiety-like behavior was also measured by the EPM test [44]. The EPM apparatus mainly consists of two closed arms and two open arms. The closed arms in the apparatus were enclosed by a black wall. Each rat was placed in the central zone of the EPM facing one of the open arms. Time of staying in the arms and number of entering arms was measured for 5 min. The EPM test was implemented on the first day after inducing the PND rat model.

### Brain tissue harvest

According to the results of the MWM test, we chose the time to collect brain tissue. Since the cognitive tests were different only on day 1 after sevoflurane exposure, we harvested the brain tissues on the first day after inducing the PND rat model. After deep anesthesia with sevoflurane, the animal brain tissue was collected immediately for WB (western blotting), RT-PCR (real-time polymerase chain reaction), ELISA (enzyme-linked immuno sorbent assay), and RNA-sequencing, which were stored in a refrigerator at -80 °C. Then, we also used saline flush and paraformaldehyde fixation of brain tissue for immunostaining. Brain frozen sections were prepared for HE (hematoxylin and eosin) and immunofluorescent staining, which were stored in a refrigerator at − 20 °C.

### RNA-sequencing

The hippocampus was used for RNA-sequencing. The cDNA library preparation was according to the BGI (China)’s standard procedures. In short, the fragment was end-repaired, dA-tailed, adaptor ligated, and then with a 4-cycle PCR program. The libraries were sequenced following the BGI’s protocols for RNA-Seq on the Illumina HiSeq 2500 platform by using the 50-bp pair-end sequencing strategy. Transcript expression levels were measured by using fragments per kilobase per million reads (FPKM). Pathway and process enrichment analysis has been carried out with the following ontology source: GO Biological Processes, GO Molecular Functions, and GO Cellular Components by Metascape (https://metascape.org/gp/index.html#/main/step1) [46]. All DEGs (differentially expressed genes) in the genome have been used as the enrichment background and were displayed in the volcano plot. Terms with a P-value < 0.01, a minimum count of 3, and an enrichment factor > 1.5 are collected and grouped into clusters according to their membership similarities. To further investigate the relationships between the terms, a subset of enriched terms has been selected and rendered as a network plot, where terms with a similarity > 0.3 are connected by edges. Protein–protein interaction enrichment analysis has been performed with the following databases: STRING [47], BioGrid [48], OmniPath [49], and InWeb_IM [50] by using Metascape. If the network contains between 3 and 500 proteins, the Molecular Complex Detection (MCODE) algorithm has been applied to examine densely connected network components. Pathway and process enrichment analysis has been applied to each MCODE component independently, and the terms by P-value have been reserved as the functional description of the corresponding components. Expression heatmaps were produced by Dor. Tom from BGI (a web-based multi-omics visualization tool: https://biosys.bgi.com/#/report/mrna/en/help).

### RT-PCR

Total RNA of the hippocampus was extracted and retrotranscribed into cDNA by using an EntiLink™ 1st Strand cDNA Synthesis Kit (ELK Biotechnology, China) according to manufacturer’s instructions. RT-PCR was performed using a StepOne™ Real-Time PCR machine (Thermo Fisher Scientific, America) and an EnTurbo™ SYBR Green PCR SuperMix kit (ELK Biotechnology, China). The reaction system was prepared according to the instruction. The cycling parameters were 95 °C for 3 min, then 40 cycles at 95 °C for 10 s, followed by 58 °C for 30 s and 72 °C for 30 s. All gene primer pairs are shown in Table 1.

**Table 1:** Primers of RT-PCR

RNA	Sequences forward/reversed 5′-3′	
β-actin	Forward	CGTTGACATCCGTAAAGACCTC
	Reversed	TAGGAGCCAGGGCAGTAATCT
PEX5R/Trip8b	Forward	CACCATCCTGTTCATGGAAGC
	Reversed	GCCATCAGAGCCTTCAAGTTG
HCN1	Forward	AGAAATGAAGTTGACAGACGGC
	Reversed	GGATATTCCTCCAAGACCTCGT
HCN2	Forward	GATGCGCATCTGTAACCTGATC
	Reversed	GCCCGTAGCCAATACAGAGC
HCN3	Forward	AGACATGGCTCGTGGTATTCG
	Reversed	TGAGCGTCTAGCAGATCGAGC
HCN4	Forward	CACCCGTAGGCATGTCTGAC
	Reversed	GTAGCGGTGTTCGTAGTAGTCGT
CD68	Forward	CAGTGGACATTCTCAGCGCA
	Reversed	GTAACGCAGAAGGCAATGAGC

### WB

Total protein was extracted from the hippocampus on the first day after inducing the PND rat model. The sample was added to lysis buffer (RIPA) with protease and phosphatase inhibitors. The tissue homogenizer was used to produce hippocampal homogenates. Afterward, homogenates were centrifuged at 4 °C at 12,000 rpm for 15 min, and the supernatants were then collected in EP tubes. The concentration of protein in EP tubes was measured by a BCA protein assay Kit (Biosharp, China). Next, protein samples were separated by electrophoresis on 8–12% sodium dodecyl sulfate–polyacrylamide gels and transferred to PVDF membranes (Millipore, America). The PVDF membranes were blocked with 5% nonfat milk in 0.1% tris-buffered saline with Tween 20 (TBST), followed by incubation overnight with a primary antibody. The following primary antibodies in the study were used, including anti-HCN2 (1/1000, Invitrogen, America), anti-PEX5R (1/1000, Santa Cruz Biotechnology, America), and anti-iba1 (1/1000, Abcam, United Kingdom). After washing with TBST, the PVDF membranes were incubated with HRP-labeled secondary antibody at RT (room temperature) for one hour. The following second antibodies in the study were used, including anti-rabbit (1/10000, Abcam, United Kingdom) and anti-mouse (1/10000, Abcam, United Kingdom). Then, ECL-A buffer and ECL-B buffer (Biosharp, China) were added for imaging. β-actin (1/5000, Abcam, United Kingdom) was employed as a loading control. The WB images were quantified using Image J software.

### Immunofluorescence (IF) staining

Briefly, microwave antigen retrieval was implemented in sodium citrate buffer (10 mM sodium citrate) at 95 to 100 °C for 10 min. Sections were blocked with 5% bovine serum albumin in phosphate-buffered saline (PBS) containing 0.3% Triton-X 100 for 1 h at RT. After washing with PBS, the brain slices were then incubated overnight at 4 °C with primary antibodies, including anti-HCN2 (1/200, Invitrogen, America), anti-Neun (1/200, Abcam, United Kingdom), anti-GFAP (1/200, Abcam, United Kingdom), and anti-iba1 (1/200, Abcam, United Kingdom). Two antibodies were co-incubated with sections to achieve bimolecular staining. Afterward, the brain slices were incubated with goat anti-rabbit IgG antibody conjugated with Alexa Fluor 488 (1/1000, Abcam, United Kingdom) or goat anti-mouse IgG antibody conjugated with Alexa Fluor 594 (1/1000, Abcam, United Kingdom) for one hour at RT in a dark room. Brain slices were mounted with Mounting Medium With DAPI—Aqueous, Fluoroshield (Abcam, United Kingdom) and then imaged under a fluorescence microscope. Immunostaining was analyzed by using the Image J software.

### ELISA

Cytokines (TNF-α, IL-6, and IL-1β) in the hippocampus and cortex were quantified by using ELISA kits (Bioswamp, China). According to the manufacturer’s instructions, an ELISA was performed. A microplate reader (Thermo Fisher Scientific, America) with absorption at 450 nm was used to measure absorbance values of samples, and cytokines concentration was detected according to the standard curve produced at the same time.

### HE staining

Brain slices were stained with an H&E kit (Solarbio, China) according to the manufacturers' instructions.

### Statistical analysis

Results are reported as mean ± SD (standard deviation) to describe data. The data between the two groups were analyzed using the t-test or rank-sum test. In RNA-sequencing results, DEGs were selected by meeting the two criteria: |log_2_(fold change, FC)|> 1 and adjusted *P* < 0.05. The fold change of each gene was calculated by comparing the standardized read counts of the PND group to the control group (fold change = standardized read counts of the PND group/standardized read counts of the control group). A *P* < 0.05 was considered as a significant difference. All data analyses were performed with Stata 16.0 (Stata Corporation, America).

## Supplementary Information

**Additional file 1: Figure S1.** Co-labeling of HCN2 with microglia, astrocytes, and neurons. Arrows indicate co-labeling of HCN2 with cells.

## Data Availability

All data generated or analysed during this study are included in this published article.

## Abbreviations

PND: Perioperative neurocognitive disorders
HCN: Hyperpolarization-activated cyclic nucleotide-gated (HCN) channels
IL-1β: Interleukin-1β
TNF-α: Tumor necrosis factor α
CSF1R: Colony-stimulating factor 1 receptor
SD: Sprague Dawley
PND-NS: PND-Normal saline
PND-HCN-B: PND-HCN channel blocker
AP: Anteroposterior
ML: Mediolateral
DV: Dorsoventral
MWM: Morris water maze
OFT: Open field test
EPM: Elevated plus maze
WB: Western blotting
RT-PCR: Real-time polymerase chain reaction
ELISA: Enzyme-linked immuno sorbent assay
HE: Hematoxylin and eosin
FPKM: Fragments per kilobase per million reads
DEGs: Differentially expressed genes
GO: Gene Ontology database
KEGG: Kyoto Encyclopedia of Genes and Genomes database
FC: Fold change
PEX5R/Trip8b: Tetratricopeptide repeat-containing Rab8b-interacting protein
SD: Standard deviation
IQR: Interquartile range
