# Supplementary figures and images for: PEX5R/Trip8b-HCN2 channel regulating neuroinflammation involved in perioperative neurocognitive disorders
Source: Cell Biosci. 2022 Sep 14;12:156. doi: 10.1186/s13578-022-00892-6 (PMC9476339; doi:10.1186/s13578-022-00892-6)

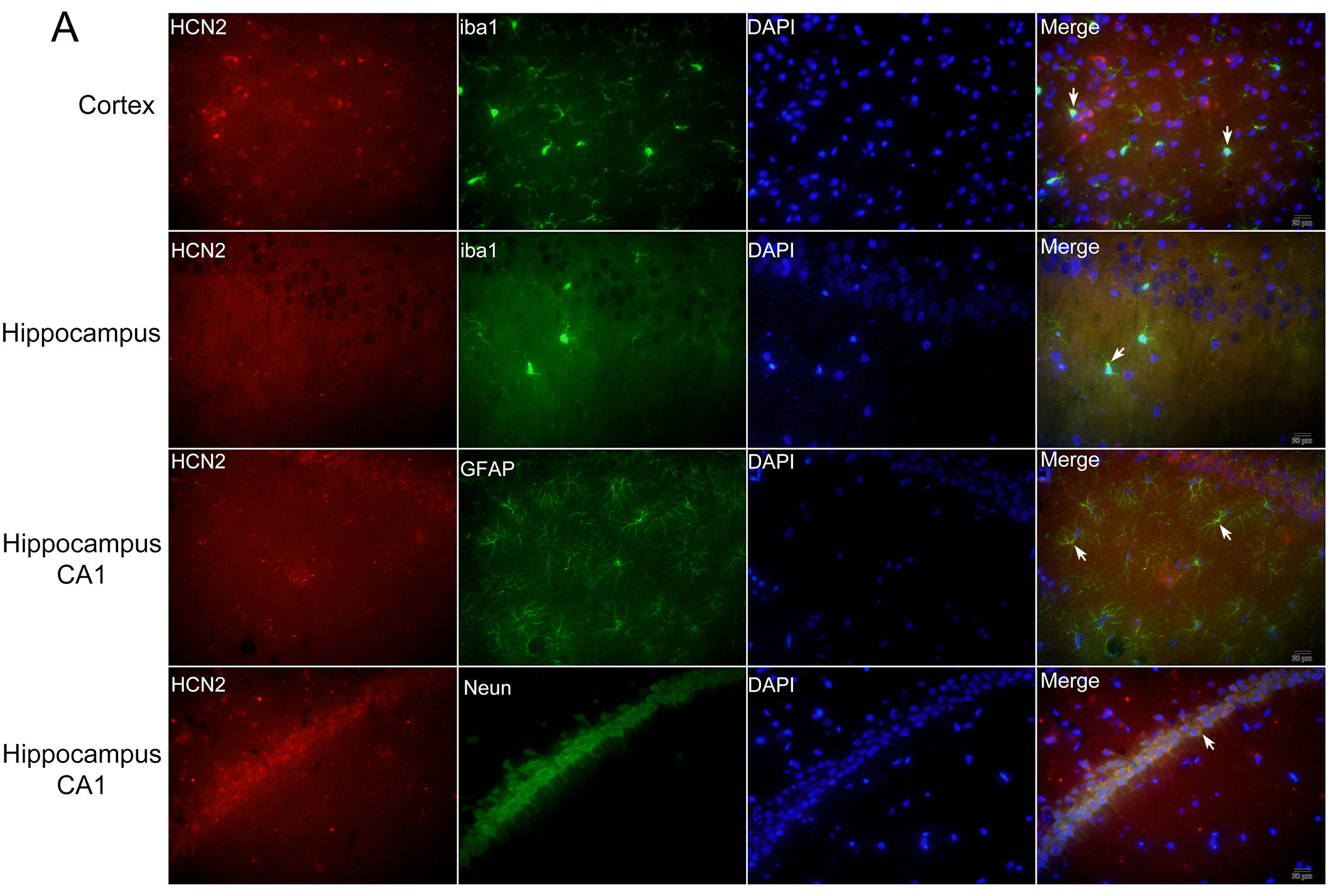

Supplement: Supplementary file 1 — Additional file 1: Figure S1. Co-labeling of HCN2 with microglia, astrocytes, and neurons. Arrows indicate co-labeling of HCN2 with cells. [file 13578_2022_892_MOESM1_ESM.jpg]
